# The relationship between released soluble FceRI-alpha and its cell surface density on human basophils

**DOI:** 10.1371/journal.pone.0245942

**Published:** 2021-01-22

**Authors:** Donald MacGlashan

**Affiliations:** Division of Allergy and Clinical Immunology, Department of Medicine, Johns Hopkins University, Baltimore, Maryland, United States of America; King’s College London, UNITED KINGDOM

## Abstract

**Background:**

The IgE-mediated activation of mast cells and basophils results in the secretion of many substances, including the release of FceRI-alpha subunit. This released alpha subunit can bind IgE and it may act as a down-regulator of subsequent IgE-dependent reactions. However, previous studies do not observe loss of the mass of FceRI-alpha associated with the cells, at least not for human basophils. This study was designed to understand the basis for the discordant observations.

**Methods:**

Purified human basophils were stimulated with multiple activating secretagogues and supernatants were examined for histamine and released FceRI-alpha. In addition, cell surface IgE densities (occupied and unoccupied) were measured by flow cytometry and total cellular content of mature and immature FceRI-alpha determined with Western blots.

**Results:**

Released FceRI-alpha, on average, represented 7% of the total surface FceRI before the reaction. The molecular weight of the soluble FceRI-alpha was approximately 54 kD, larger than immature subunit and somewhat smaller than surface subunit. In addition, 1) release ceased long before internalized FceRI-alpha was processed, 2) release was insensitive to Bafilomycin A, 3) release was independent of the starting density of FceRI and 4) release occurred more effectively with non-IgE-dependent stimuli, FMLP or C5a.

**Conclusions:**

There appears to be relatively constant amount of nearly mature FceRI-alpha that is susceptible to secretion events induced by any form of stimulation. The amount, on average, represents about 7% of the mature form of FceRI-alpha.

## Introduction

IgE-mediated activation of basophils and mast cells results in the secretion of substances, e.g., histamine, that generate the signs and symptoms of the allergic response. There are many “secreted” products that are not well studied but one that has generated some interest is the secretion of the high affinity IgE receptor itself. Studies by Fiebiger and colleagues [1–3] have demonstrated the presence of the alpha subunit of the receptor in the serum of subjects and this group has also shown the appearance of an alpha subunit in the supernatants of an *in vitro*-stimulated mast cell line. In both cases, this solution-phase alpha subunit is able to bind IgE and the investigators have proposed that it can act as a feedback inhibitor of further allergic reactions because it becomes a “sink” for binding IgE that prevents its ability to sensitize cells expressing the high affinity receptor.

**Fig 1:**
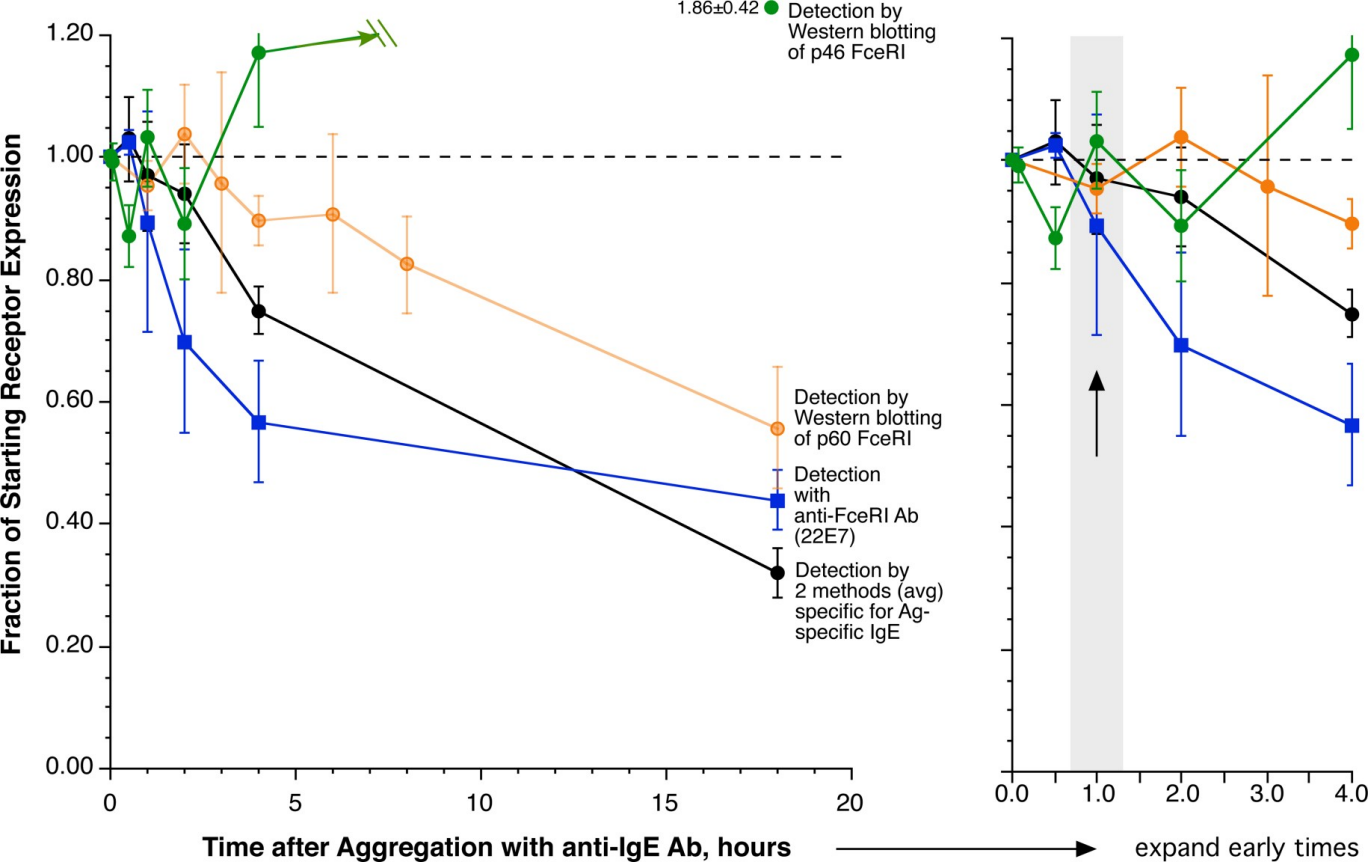
Behavior of surface FceRI following IgE-mediated stimulation of human basophils. The data is derived from former publications [5]. Panel A summarizes several different methods used to detect cell surface FceRI by flow cytometry (black and blue symbols) or Western blotting using monoclonal antibody 22E7 to detect both forms of FceRI-alpha (green and orange symbols). Panel B expands on the early times with the grey region signifying the period of stimulation for the ELISA measurements of released FceRI-alpha.

An issue arises, however, for understanding how this occurs given that previous *in vitro* studies [4, 5] have also observed that during activation of basophils or mast cells that FceRI alpha subunit is not lost from the cell surface during the time frame of the reactions that the Fiebiger studies explore. For example, **Fig 1**summarizes previous work in human basophils. It is notable that by neither flow cytometry to detect surface IgE or FceRI or by western blots for detecting both surface FceRI (p60) and internal FceRI (p46, a glycosylation- pre-golgi- immature form of FceRIalpha) does one observe loss of FceRIalpha after one hour of stimulation, or indeed, statistically, not for several hours. In fact, internal FceRIalpha, p46, increases, as assessed by western blotting.

We hypothesized that the amount of released FceRI-alpha might be but a small fraction of the available cellular FceRI-alpha and its loss in the various assays employed is too small to be observable with flow cytometry or western blot assays.

## Methods

### Materials

The following were purchased: PIPES, BSA, EDTA, D-glucose, erythrosin B (Sigma-Aldrich, St. Louis, MO, USA); anti-mouse IgG1-Alexa Fluor 647, human IgG (Cappel Laboratories, Malvern, Pa); anti-FceRI, 15A5, and 22E7 (gift from Hoffman-LaRoche); human IgG (Cappel Laboratories); recombinant C5a (#4995B, BioVision, Milpitas, CA); FMLP (formyl-met-leu-phe, Sigma, St. Louis, MO).

### Buffers

PAG buffer was prepared with 25 mM PIPES, 110 mM NaCl, 5 mM KCl, 0.1% glucose, and 0.003% HSA. PAGCM was PAG supplemented with 1 mM CaCl_2_ and 1 mM MgCl_2_. PAG-EDTA (ethylenediamine N, N, N’, N’- tetra-acetic acid) consisted of PAG supplemented with 4 mM EDTA, elutration buffer, PAG containing 0.25% BSA. Phosphate buffered saline (PBS) was purchased as a 10X solution, Nuclease-free H_2_O (available from various molecular biology suppliers). Lactic acid IgE elution buffer 0.01 M lactic acid, 0.14 M NaCl, 0.005 M KCl at pH 3.9 [6].

### Cell purification

Peripheral blood basophils were enriched to purities >98% through the combined use of 2-step Percoll gradients and negative selection using the basophil reagents from StemCell Technologies and columns from Miltenyi, as described in previous publications [7]. The average purity of these basophils by alcian blue staining [8] was 99%. Starting viability of these cells was typically >97%.

### Removal of endogenous IgE and histamine release

For some experiments, a portion of the endogenous IgE was removed by treating the cells with lactic acid buffer (on ice for 6 seconds with buffer before adding 1.5 ml EB to neutralize the pH) [5]. Histamine release was measured from supernatants of cells stimulated in PAGCM buffer. Histamine was measured by automated fluorimetry [9].

### Analysis of surface markers by flow cytometry

Flow cytometric measurements were performed on a BD Accuri flow cytometer. Cells were fixed in 2% paraformaldehyde for 20 minutes at room temperature, and the cell suspension was stored at 4˚C. Cells were blocked with 0.2 mg/ml non-specific human IgG and labeled first with either nonspecific goat IgG, nonspecific mouse IgG1, goat anti-hIgE antibody, 22E7, or 15A5 Abs at concentrations previously determined to be optimal for flow cytometry (25 minute incubation) [10]. A second 25-minute incubation with anti-mouseIgG1-alexa647, or anti-goat-alexa647 with anti-CD123-FITC and CCR3 (PE-labeled) (1/30 dilution from manufacturer’s stock solution) was followed by washing and analysis.

### Western blot analysis

Basophils were lysed in 1X ESB (electrophoresis buffer, Novex Tris-Glycine SDS sample buffer; Invitrogen, Carlsbad, CA) at a density of 300,000/20 mL of ESB, boiled for 5 minutes, and stored at -80˚C until SDS-PAGE analysis. Western blotting was performed in a semi-quantitative manner; briefly, 3 dilutions (1.0, 0.4, 0.15 where 1.0 = undiluted lysate) of a standard preparation of basophils were run with samples in 15-lane 10% gels, with molecular weight markers in the first and last lanes [10]. After transfer to nitrocellulose, the membrane was blotted for FceRI-alpha with 22E7 monoclonal Ab. For changes in receptor expression, lane-loading was also checked by blotting with anti-p85 antibody (p85 subunit of phosphoinositide 3-kinase) that we have previously shown to be an excellent protein for normalizing the between-lane results. The band densities were determined by digital scanning and converted to absolute cellular densities using the internal standard where the integrated p60 is equal to flow cytometrically determined cell surface expression density (previous studies have demonstrated that the p60 accurately represents the plasma membrane associated FceRI-alpha [10]).

### ELISA for soluble FceRI-alpha

The commercially available ELISA assay (ThermoFisher, Rockville, MD) for human FceRI-alpha was used to quantitate samples. This standard for this assay has been calibrated and validated.

## Results

### Relationship of soluble FceRI-alpha to cellular FceRI-alpha

To test the primary hypothesis required quantitative assessments of several parameters of the cells being stimulated. In these studies, human peripheral blood basophils, purified to >98%, were used as the test model and the cells were stimulated with an optimal concentration of anti-IgE antibody for 1 hour. Surface bound IgE, and surface FceRIalpha were assessed by flow cytometry using a calibrated assay developed for multiple previous studies [10]. Histamine release was the metric to assess the strength of activation through the IgE receptor. To capture the total cell content of both the mature cell surface FceRI (a p60 band) and the immature form (a p46 band that is strictly localized internally and pre-golgi processed receptor), semi-quantitative [10] Western blots of cell lysates before and after stimulation were performed. The supernatants were also analyzed for solution phase FceRIalpha using a commercial ELISA kit used for previously published studies of this molecule [11]. This kit has a calibrated standard to provide for quantitation of the solution-phase FceRIalpha. Provided this standard is accurate, the assay provides an estimate of the molecules of FceRIalpha released by each cell.

**Fig 2:**
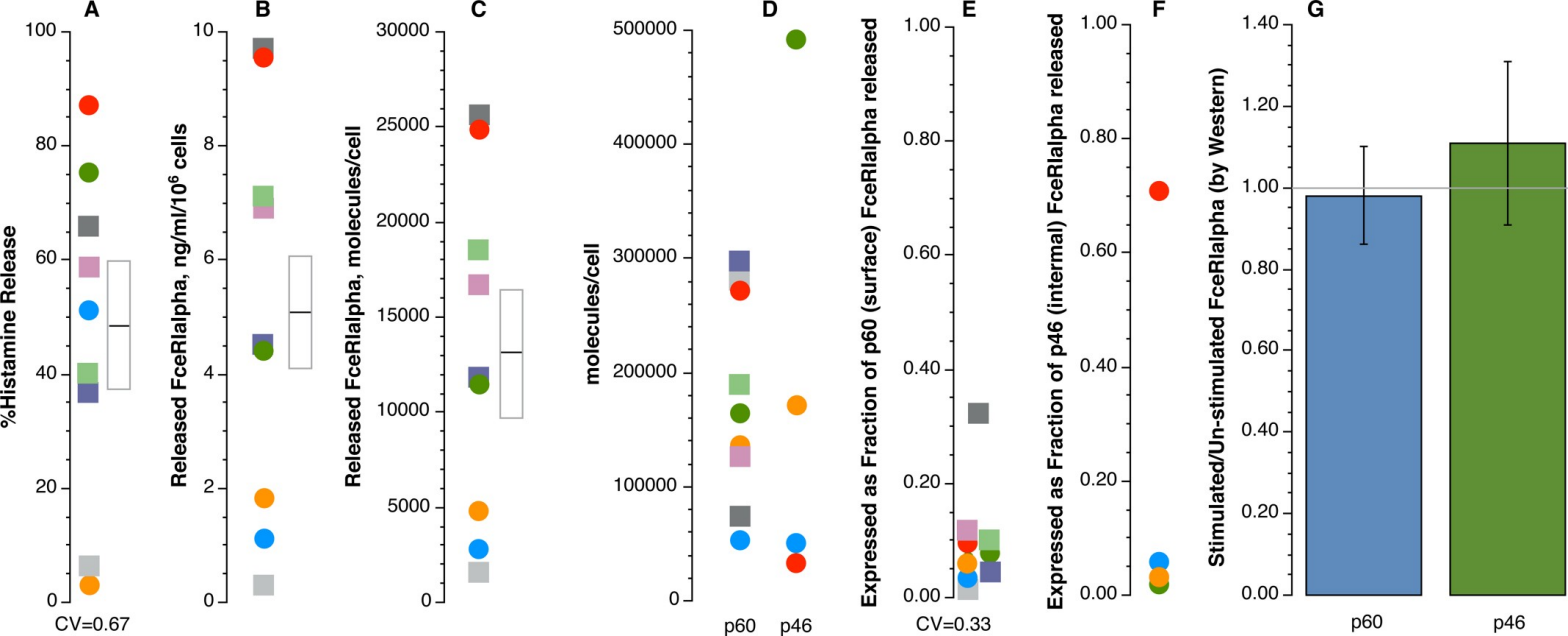
Summary of basophil characteristics for the various resting and stimulated metrics. Each symbol represents a different preparation of purified basophils. For all preparations, the stimulation used the same concentration of monoclonal anti-IgE Ab for a 1-hour incubation before recovering supernatants and pellets. Data analysis for the samples signified by the circular symbols represents samples also analyzed by Western blot (i.e. the far right panel represents n = 4 preparations). For release experiments, basophil counts per condition were made in order to make the normalizing calculations shown. Panel A: net percent histamine release from the various experiments, the mean ± standard error of the mean (SEM) is shown by the box and bar to the right of the data points. Panel B: net released FceRIalpha from the same experiments shown in panel A (box and bar showing the mean ± SEM). Panel C: data from panel B re-cast in terms of molecules of released FceRIalpha per cell (box and bar showing the mean ± SEM). Panel D: resting densities of surface FceRIalpha (p60) as measured in calibrated flow cytometry and in 4 experiments, the density of internal pre-golgi FceRIalpha (p46) calculated by comparing the Western blot band densities of p60 vs. p46 forms from un-stimulated cells and using the calibrated flow cytometry results to calculate the p46 density. Panel E: combines the results from panel C with panel D (p60) to represent the released FceRIalpha as a fraction of the surface FceRIalpha. Panel F: similar to panel E but using the calculated internal (p46) results to calculate the released FceRIalpha as a fraction of internal p46 density (n = 4). Panel G: Using Western blot results from 4 experiments to determine whether there was loss of p60 or p46 forms of FceRIalpha following stimulation (to re-capitulate earlier study results [5]).

**Fig 2**summarizes our results from 4–9 experiments (all measurements were made with 4 preparations and 5 additional preparations were examined without western blotting). Released FceRIalpha represents approximately 7% (median) of the original surface FceRIalpha. Western blots of these samples do not show any apparent loss of either p60 or p46 FceRIalpha, a result consistent with prior published reports [5]. However, as can be seen in the prior observations shown in **Fig 1**, a 5–10% loss of surface FceRIalpha would lie within the noise of the experiments for a one-hour release period. This result potentially reconciles the original quantitative question about FceRI-alpha release.

**Fig 3:**
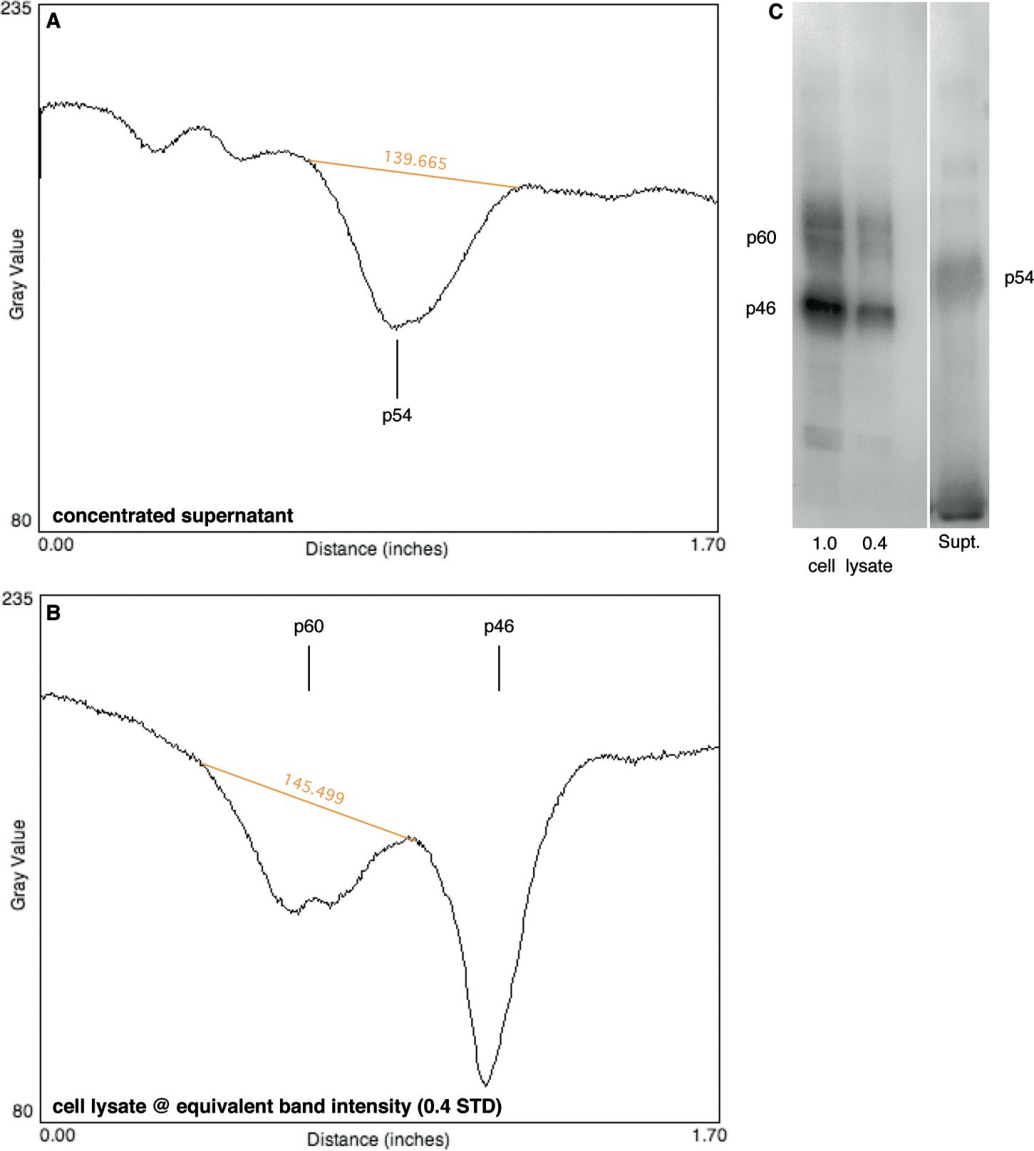
Western blot of cell lysates and released FceRI-alpha. FceRI-alpha was detected in the semi-quantitative Western blots using 22E7 monoclonal Ab. A basophil preparation with a known surface density of FceRI was used as a standard from which 3 dilutions were used in the gel electrophoresis in order to provide a titration curve and estimate the quantity of FceRI-alpha detected [5]. Two of the standard cell lysate dilutions are shown in panel C. The far right lane represents a concentrate of the released FceRI-alpha obtained by adsorption of the supernatant on an IgE-sepharose bead followed by elution with boiling electrophoresis buffer prior to running the gel. Panels A and B profile the digital scan traces for the far right lane (panel A) and the far left lane (panel B). Molecular weight markers (not shown) locate the position and size of the bands.

The western blotting results show that the released FceRI-alpha has a molecular weight of approximately 54 kD. The heterogeneity (breadth of the band) is similar to p60 and broader than p46, which is typically a narrow band (see **Fig 3**). Running the supernatants directly in the Western revealed an *absence* of identifiable bands. However, concentrating the supernatant by adsorption with an IgE-coupled-sepharose bead by 14-fold allows the protein to be visualized. This is consistent with the ELISA assay showing levels that could not be visualized in the Western blots without concentration. The molecular weight is similar to release from the transfected MelJuso cell line [1].

### FceRI-alpha release characteristics

The focus of this study was not to identify the source of the released alpha. But there were some issues that were explored while performing these experiments that spoke to the question of source and the limits of its production. In its native design, the assay detects total soluble FceRI-alpha, i.e., receptor with or without bound IgE. Eliminating the second incubation of the assay protocol (the IgE binding step) measures the fraction of soluble FceRI-alpha that already has IgE bound. While not all the samples were measured this way, of those that were, only 10% of the total soluble receptor had pre-bound IgE, i.e., 90% of the soluble receptor was unoccupied. It is reasonable to ask whether a receptor that is not involved in the crosslinking reaction is being processed during activation. Prior studies have suggested that uninvolved receptors are not internalized and if only cross-linked receptors are internalized, then the appearance of released unoccupied receptor suggests endosomal processing. Four lines of testing should help examine this possibility.

**Fig 4:**
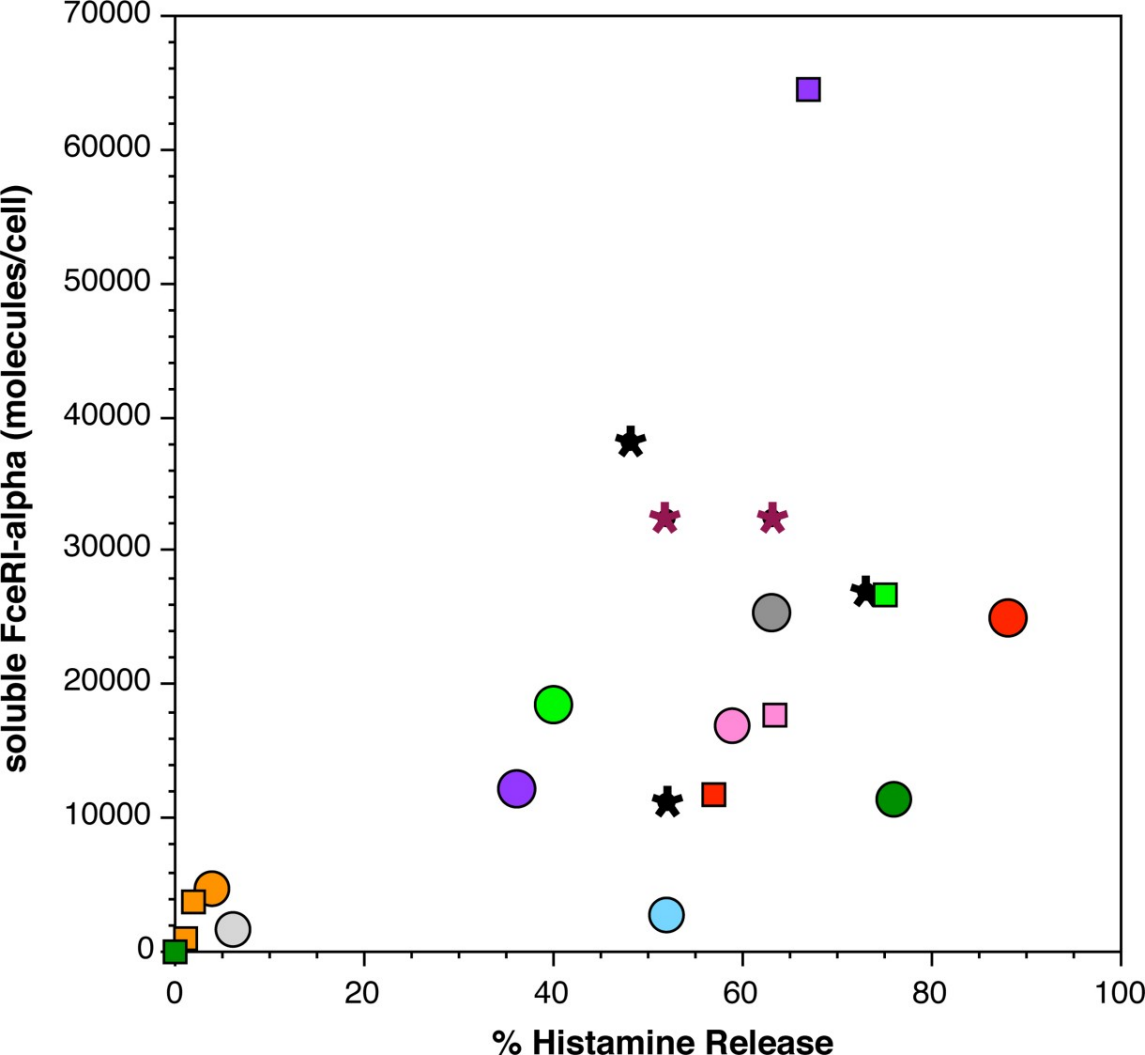
Correlation between stimulus-induced histamine release and the release of soluble FceRI-alpha (expressed as number of molecules released per cell). Circle symbols (different colors) represent different preparations of basophils (the colors match with those in Fig 2). Square symbols represent different levels of stimulation for a given preparation where the color matches the result with the 1 μg/ml anti-IgE Ab response (represented by the round symbol). Star symbols represent either FMLP- (n = 3) or C5a- (n = 2) induced release.

**Fig 5:**
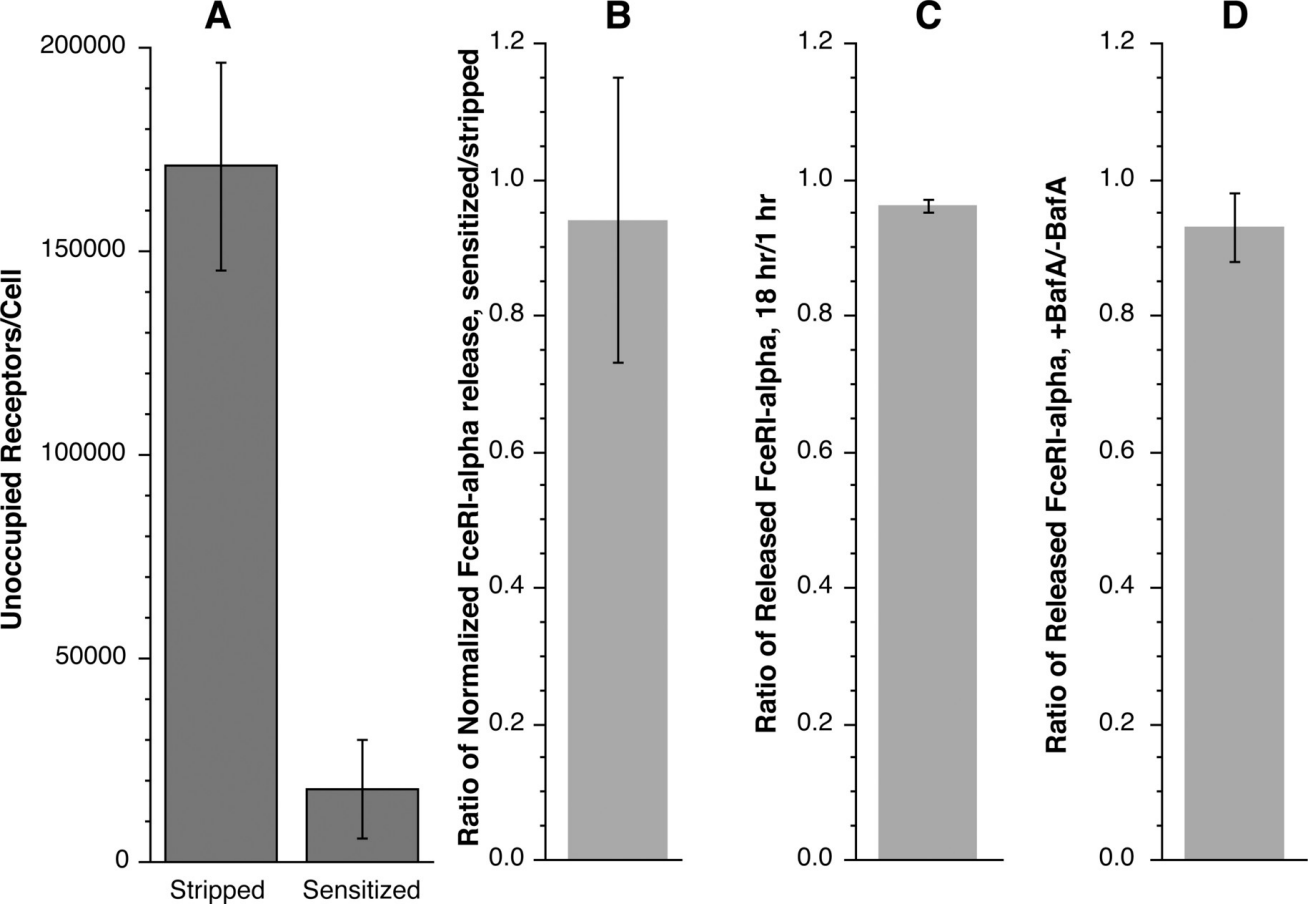
Release ratios with under different conditions. Panel A/B; basophils were treated with lactic acid buffer to remove a significant fraction of endogenous IgE, one-half were then re-sensitized with IgE, both were stimulated with 1 μg/ml anti-IgE and released FceRI-alpha measured. A portion of the cells were analyzed by calibrated flow cytometry for unoccupied receptor density (panel A). Released FceRIalpha was normalized by the amount of histamine release (which differs somewhat due to the stripping procedure, panel B). Panel C; ratio of released alpha from 1-hour and 18-hour cultures. Panel D; ratio of released FceRI-alpha in the presence or absence of Bafilomycin A.

First, the amount of released FceRI-alpha might be partially dependent on the starting surface FceRI density. There was considerable variability in the amount of released FceRI-alpha subunit (see **Fig 2B and 2C**). However, the amount of histamine release (a metric of the strength of activation) was likely the primary determinant (Rs = 0.69, p = 0.035, for only the inter-preparation samples, circle symbols in **Fig 4**). For several of these experiments, there were other conditions tested to assess the potential role of unoccupied receptors. In some experiments, endogenous surface IgE was dissociated with lactic acid to generate more unoccupied receptors, testing whether more unoccupied receptors would produce more soluble (unbound) FceRI-alpha. There was no relationship found with this experimental maneuver (**Fig 5A and 5B**).

But these conditions led to suboptimal histamine release with concordant reductions in released alpha subunit. In other experiments, different concentrations (1 μg/ml vs. 0.1 μg/ml) of the stimulating anti-IgE Ab also produced different histamine release and proportional soluble FceRI-alpha release (suboptimal/optimal HR = 0.49±0.11; suboptimal/optimal sFcR = 0.47±0.13; another way to view this, the relationship between suboptimal and optimal release for the two outcomes showed and Rs = 0.80, p = 0.02). **Fig 4**shows all the results in a format that allows a comparison of both inter-preparation differences and intra-preparation differences in stimulation (in **Fig 4,** square symbols represent these suboptimal levels of stimulation (generated by different methods, stripping (see below) or dilution of the stimulus) with color connecting the results for a given preparation). In other words, the intra-experimental stimulus-dependent differences and inter-preparation variability both suggested that the strength of the response was a primary determinant of the amount of released FceRI-alpha. A two-factor analysis that incorporated the density of surface total FceRI (range of 45,000/cell to 299,000/cell) (or alternatively, unoccupied FceRI) into the inter-preparation analysis suggested no role for density in predicting the amount of released alpha.

For a second line of testing, if processing of internalized alpha occurs (with subsequent secretion), the time course shown in **Fig 1**would suggest that additional released FceRI-alpha should occur by 18 hours (where previous studies show a loss of 50% of the total surface FceRI mass, see **Fig 1**). However, comparing 1 hour to 18 hours showed no additional released FceRI-alpha (18hr/1hr = 0.96±0.01, n = 2) (**Fig 5C**). For these experiments, cells were incubated in RPMI-1640 media containing 0.03% HSA and supplemented with calcium to bring its concentration to 1 mM. The incubation time was 1 or 18 hours. For comparison to the experiments in Fig 2, an aliquot of cells was also incubated in PAGCM buffer for 1 hour. There were no differences in release between the two one-hour conditions. Average FceRIalpha release was 2.4 ng/ml at one hour and 2.3 ng/ml at 18 hours.

For a third test series, since most surface FceRI is occupied by IgE [12] and 90% the released alpha is unoccupied, then it stands to reason that IgE should be dissociated from internalized FceRI prior to secretion. Previous studies of FceRI processing have shown that it is sensitive to acidification inhibitors like Bafilomycin A [5]. However, comparing released FceRI-alpha ± 200 nM Bafilomycin A shows no difference (ratio of +BA/-BA = 0.93±0.05, n = 2) (**Fig 5D**). The same two experiments used for the 18-hour test above included Bafilomycin A in the one-hour test point (average release without Bafilomycin A was 2.4 ng/ml and 2.2 ng/ml with Bafilomycin A).

Finally, these results suggest a process that is not related to internalization. Further evidence that this is true is that stimulation with the non-IgE receptor-dependent stimulus, FMLP or C5a, which are GTP-protein dependent receptors. Both stimuli also cause the release of FceRI-alpha at levels similar to stimulation with anti-IgE Ab (FMLP-induced histamine release = 58%, soluble FceRI-alpha release = 25,400/cell; C5a-induced histamine release = 58%, FceRI-alpha release = 32,300/cell; compare to the average anti-IgE Ab-induced histamine release of 49% and soluble FceRI-alpha release of 12,600/cell). **Fig 4**shows the FMLP and C5a results as star symbols. As a fraction of surface FceRI, FMLP and C5a released somewhat more FceRI-alpha per cell.

## Discussion

During secretion from basophils, FceRIalpha appears in the supernatant but it represents only a small percentage (median = 7%, range of 0.004% - 34%, see **Fig 2E**) of the original surface FceRI-alpha. The molecular weight (p54) is greater than the internal stores of FceRI (p46) and less than the surface FceRI (p60) suggesting that the source is not p46 protein. However, it may represent a break-down product of surface p60 or unfinished maturation of the p46.

The primary purpose of this study was to resolve the discrepancy between previous studies showing no loss of cell-associated FceRI-alpha during the first hour stimulation and the appearance of soluble FceRI-alpha. The quantitative data speaks to this discrepancy. However, the results also highlight the question of the source of FceRI-alpha and while this study did not explore this question extensively, some of its results do speak to the question.

The results show several important points. Release occurs only during the first hour of secretion and not later when western blot results show the primary loss of FceRI-p60 mass. Furthermore, release is insensitive to an endosomal acidification inhibitor. Prior studies in a transfected cell showed that chloroquine inhibited release [1] but chloroquine is not an acidification inhibitor, instead preventing fusion of internal vesicles [13] (e.g., autophagasome to the lysosome). Finally, a non-IgE dependent stimulus also induces release of FceRI-alpha.

Four explanations merit consideration; 1) soluble FceRI-alpha represents a processed product of internalized surface FceRI, 2) soluble FceRI-alpha represents shedding of the plasma membrane, 3) soluble FceRI-alpha represents release of an occult pool of FceRI not yet placed on the cell surface and 4) soluble FceRI-alpha represents a enzymatically “clipped” surface FceRI. Each of these explanations suffers from counter-expectations.

1) As noted, 7% of surface alpha is small enough to be difficult to observe, making this possibility viable. However, the majority of processing internalized alpha occurs between 1 and 18 hours and no further soluble FceRI-alpha is released in this time interval. Furthermore, treatment with Bafilomycin A had no effect. Finally, non-IgE-dependent stimuli cause release of soluble FceRI-alpha and these stimuli do not induce internalization of surface FceRI.

2) Degranulation, and the attendant plasma membrane changes, results in micro-particle release of the plasma membrane. Micro-particles expressing FceRI have been found in serum of subjects and electron-microscopic studies observe considerable membrane fragments surrounding degranulating basophils[14]. The counter-argument to this explanation is that it might be expected that cell surface density influences the amount of released soluble FceRI-alpha and this was not found to occur. In addition, studies by Dehlink et al. demonstrated the ultra-centrifugation does not alter the measurable soluble FceRI [1].

3) A small pool (7%) of nearly mature p60 FceRI-alpha that has not been placed on the cell surface, a pool that is susceptible to excretion during the complex granule fusion reaction that occurs in these cells regardless of the stimulus. While comparisons of the Western blot-determined p60 and flow cytometrically-determined p60 track closely [10], these assays are not precise enough to discriminate a 7% discordance in these measurements. The amount of ‘yet-to-be-surface-loaded’ FceRI-alpha might be relatively constant for cell preparations, leading to the apparent variability in the fraction of surface FceRI-alpha released (see **Fig 2E**). There is a modest inverse correlation between the density of surface FceRI-alpha determined by flow cytometry and the fraction of surface alpha released (these are related variables, so a correlation calculation is not valid), suggesting that a ‘not-yet-loaded’ store is relatively constant among preparations (i.e., a fixed amount of not-yet-loaded FceRI-alpha, would produce this relationship). For example, if that ‘yet-to-loaded’ FceRI-alpha were a constant 25,000 per cell, and assuming 50% histamine release, then with only 50,000 surface FceRI, one would see an apparent 25% of the surface FceRI released but with 300,000 surface FceRI, see an apparent 4% released. In this explanation, it is simply that all cells carry a constant unloaded-mature FceRI-alpha that is susceptible to excretion. A counterargument is that we have never observed a p54-sized FceRI-alpha in whole cell lysates, notably not in cells expressing low levels of surface density where one would “tune” the Western blot exposure to properly visualize the p60 band (for example, see [10]) and therefore a p54 band with roughly half the mass should be observed. It is not (as in the above example), suggesting it doesn’t pre-exist.

4) Some portion of the surface p60 is “clipped” from its membrane anchor. Removal of the transmembrane and small internal peptide portions of the subunit would reduce its molecular weight approximately 6 kD, an amount consistent with the difference between the surface-bound form and the soluble form. Like the microparticle explanation, one would expect that surface density would be a significant factor in the amount of detected soluble FceRI-alpha. In previous studies, the MMP (membrane metalloprotease), TAPI-2, did not inhibit release of soluble FceRI-alpha, although this is only one potential MMP inhibitor to test. Furthermore, since >80% of surface FceRI is bound to IgE, we should have observed >80% of the soluble bound to IgE. Clipping could occur only to unoccupied receptor but this would imply a receptor not involved in the aggregation reaction is the target.

The quantities released do provide some boundaries on how much soluble FceRI could be available to bind to extracellular IgE. A simple calculation is that if all mast cells and basophils were fully activated (100% HR) in a 70 kg subject, that extracellular levels of FceRI-alpha could reach 39 pM (0.4 million mast cells/g of soft tissue [15, 16] * ***12*,*000*** sFceRI/cell [17, 18] ÷ 14 L of extracellular fluid) (this report’s results, **Fig 2C**, define the 12,000 value). Typical circulating IgE levels reach 930 pM (80 kIU/L [19]) in non-atopic subjects. Therefore, a maximum response can only generate approximately 4% the soluble FceRI needed to remove all IgE antibody. This difference may also explain why serum measurements of soluble FceRI-alpha always show fully occupied receptor [2, 20]: once released unoccupied, serum IgE would bind. A potentially important caveat is that the released receptor may not distribute to the entire potential volume of interstitial fluid but act in very localized ways. Furthermore, on a practical level, the release of FceRI-alpha may yet provide a useful biomarker of pre-existing mast cell or basophil activation *in vivo* [3].

## Supporting information

S1 Raw image (PDF)
