# Supplementary material for: The relationship between released soluble FceRI-alpha and its cell surface density on human basophils
Source: PLoS One. 2021 Jan 22;16(1):e0245942. doi: 10.1371/journal.pone.0245942 (PMC7822313; doi:10.1371/journal.pone.0245942)
Supplement: S1 Raw image — (PDF) [file pone.0245942.s001.pdf]

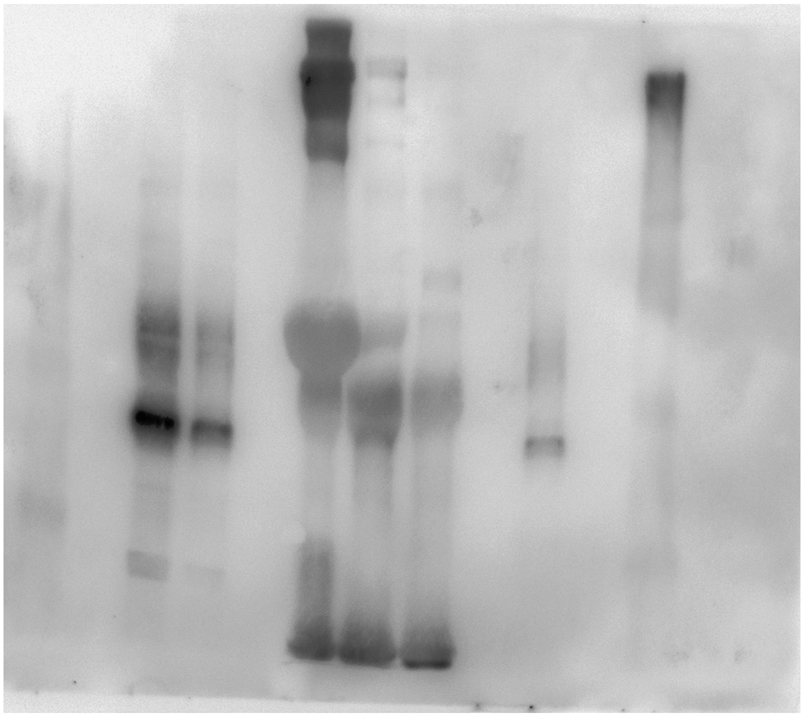

numbered lanes = those used in figure 3 (left to right)
SB = SeaBlue markers, not visible on chemiluminescence
x = lanes not used, = other standards
